# Uterine artery impedance during the first eight postpartum weeks

**DOI:** 10.1038/srep08786

**Published:** 2015-03-05

**Authors:** L. Guedes-Martins, A. R. Gaio, J. Saraiva, A. Cunha, F. Macedo, H. Almeida

**Affiliations:** 1 Department of Experimental Biology, Faculty of Medicine, University of Porto, 4200-319 Porto, Portugal; 2 IBMC-Instituto de Biologia Molecular e Celular, 4150-180 Porto, Portugal; 3 Hospital Centre of Porto EPE, Department of Women and Reproductive Medicine, Largo Prof. Abel Salazar, 4099-001 Porto, Portugal; 4 Department of Mathematics, Faculty of Sciences, University of Porto, 4169-007 Porto, Portugal; 5 CMUP-Centre of Mathematics, University of Porto, 4169-007 Porto, Portugal; 6 Department of Cardiology, Faculty of Medicine, University of Porto, 4200-319 Porto, Portugal; 7 Obstetrics-Gynecology, Hospital-CUF Porto, 4100-180 Porto, Portugal

## Abstract

The aim of this study was to construct reference ranges for the uterine artery (UtA) mean pulsatility (PI) and resistance (RI) indices from 1–8 weeks postpartum. A prospective, cross-sectional, and observational study was performed with 320 healthy women from week 1 through week 8 postpartum. UtAs were examined transvaginally using colour and pulsed Doppler imaging, and the means of the right and left values of the PI and RI, as well as the presence or absence of a bilateral protodiastolic notch, were recorded. The 5^th^, 50^th^ and 95^th^ reference percentile curves for the UtA-PI and UtA-RI were derived using regression models. The adjusted reference intervals uncovered a convergence trend at the week 8 time-point, although impedance was lower at the week 1 time-point in multiparous women compared with primiparous women. The notching prevalence was 22.5% (9/40) at week 1 and 95.0% (38/40) at week 8. The study revealed consistent evidence of a progressive increase of postpartum uterine impedance and provided new average UtA-PI and UtA-RI reference charts for weeks 1 through 8. Multiparity does not change the trend but does impart a lower rate of increase, likely as a consequence of previous vascular structural and functional differences.

Doppler ultrasound has been used to measure the flow resistance indices of the uterine arteries during the menstrual cycle^1^,^2^, pregnancy^3^, and labour^4^,^5^. Throughout the menstrual cycle, a lack of significant uterine artery (UtA) impedance changes^2^ or higher UtA impedance early and late in the cycle, compared with reduced levels mid-cycle or in the luteal phase, has been reported^6^,^7^.

The results that accompany the pelvic circulatory changes necessary to adapt the blood supply to the nutritionally demanding developing foetus are more consistent over the course of pregnancy. This unique situation translates into a continuous reduction in uterine artery resistance starting by week 6 of pregnancy^8^,^9^. This downward trend has been proposed to result from an appropriate trophoblast invasion of the uterine spiral arteries that, when abnormal, correlates with resistive uterine artery behaviour^3^,^10^.

In contrast to the extensive literature available during pregnancy, Doppler research on the uterine artery during the postpartum period is scarce. Nevertheless, puerperal conditions affecting the uterine circulation might occur, and the ability of UtA impedance, as measured by Doppler ultrasound, to predict these conditions would be immensely useful^11^,^12^,^13^. Among the potential complications, preeclampsia, postpartum haemorrhage, retained placental tissue, and infection are particularly relevant.

The absence of reference curves for uterine arteries resistance indices in an uneventful postpartum period has limited the assessment of puerperal conditions, as well as research progression in the area. This is important because in addition to providing relevant knowledge on the return of the local circulation to the previous, non-pregnant state, those reference values could help to more accurately interpret the pathophysiology of the puerperium^13^. This need led us to measure the UtA mean pulsatility (PI) and resistance (RI) indices at 1–8 weeks postpartum in an appropriate selected population to derive normative, weekly based reference ranges.

## Results

**Figure 1:**
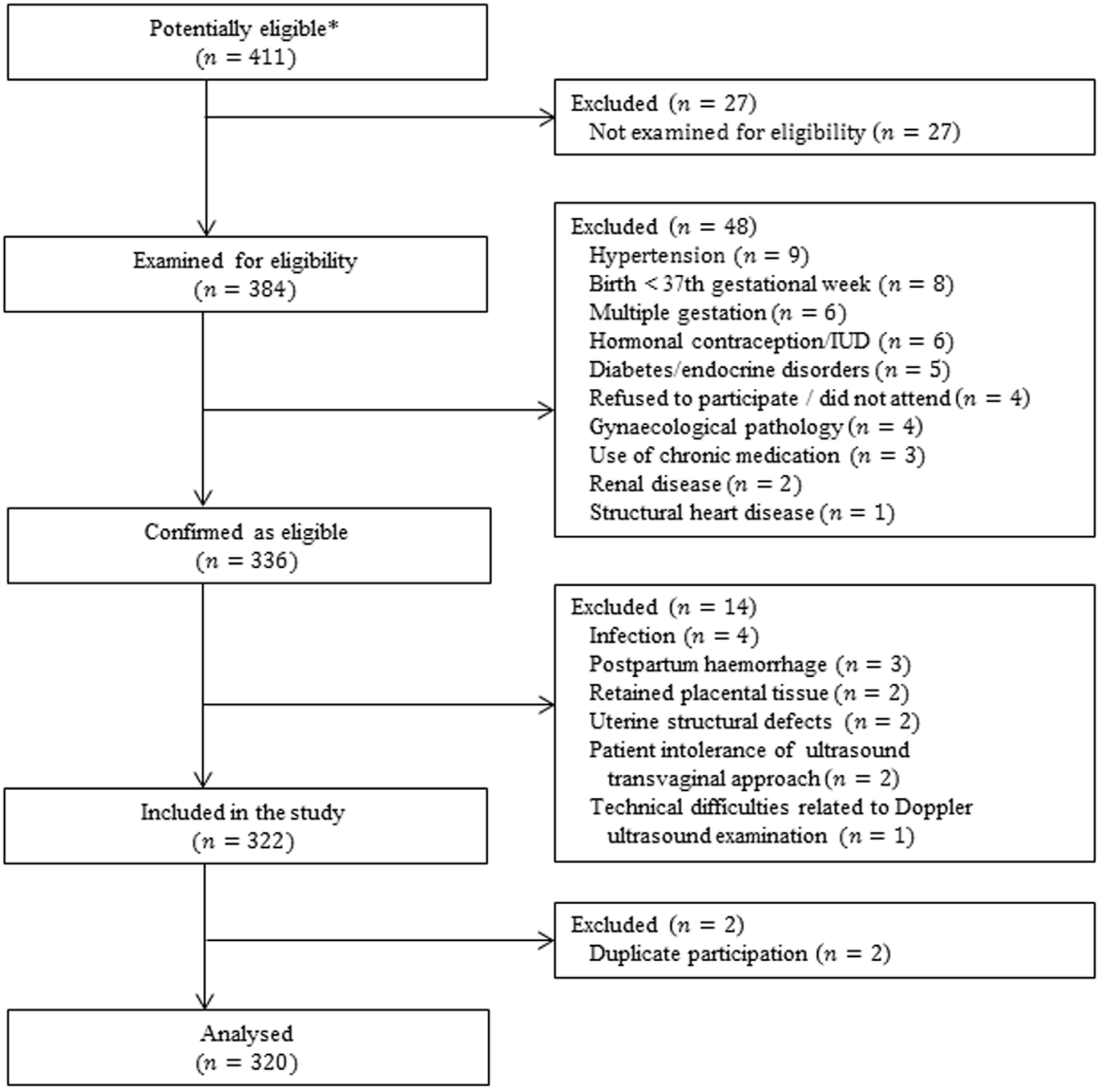
Study flowchart: numbers of postpartum women at each stage of the study. IUD, intrauterine device. * Any postpartum women attended by our clinical investigator during the study protocol were considered to be potentially eligible.

Figure 1 shows the numbers of postpartum women at each stage of the study. The main characteristics and pregnancy outcomes of the 320 women are shown in Table 1. In 40.3% (CI_95%_: 34.9–45.9) of the cases, Caesarean section was the mode of delivery; in more than 90% of those cases, the reason for that procedure was prior Caesarean delivery, dystocia, foetal distress, or breech presentation (Table 2). The evaluated postpartum period ranged from 1 to 8 weeks, and the collected data were balanced, with 40 observations made per week.

At the time of the Doppler measurements, 30.3% (CI_95%_: 25.4–35.7) of the mothers were not breastfeeding, 16.6% (CI_95%_: 12.8–21.2) were smokers, and 23.4% (CI_95%_: 18.9–28.5) had a BMI greater than or equal to 30 Kg/m^2^. The 10^th^ percentile of the sample for birth weight was 2770 g.

**Figure 2:**
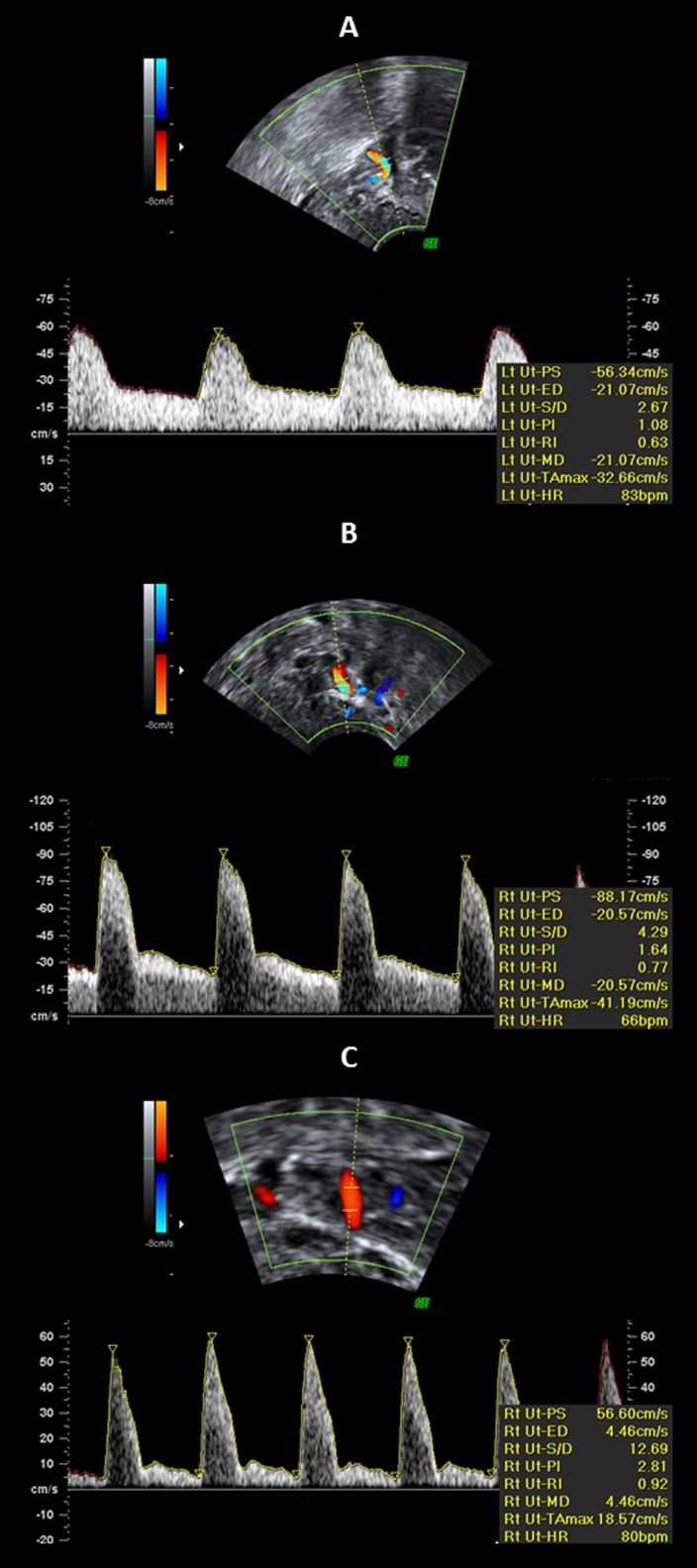
Doppler shift spectra recorded from the uterine arteries at 1 (A), 4 (B) and 8 (C) weeks postpartum. (A) Waveforms with velocities gradually decreasing from the systolic peak and with continuous forward flow in diastole; (B + C) waveforms with a notch and with continuous forward flow in diastole. The pulsatility index (PI) is used as a measurement of impedance of the flow blood distal to the sampling point and is automatically calculated according to the formula 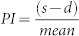, where *s* is the peak, *d* is the minimum, and the *mean* is the average maximum Doppler shift frequency over the cardiac cycle. The resistance index (RI) is automatically calculated using the formula 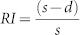, where *s* is peak systolic, *d* is end-diastolic, *c* is early diastolic, and *x* is maximum diastolic frequency.

Figure 2 shows the different types of uterine artery Doppler shift spectra obtained at different time-points. A gradual increase in the RI and PI were observed from A (week 1) to C (week 8), whereas the diastolic flow became weaker, exhibiting a well-defined protodiastolic notch (Figure 2C).

The prevalence of uterine artery positive notching (defined as at least one notch) was 22.5% (9/40) at week 1 and 95.0% (38/40) at week 8 (p < 0.001), as shown in Table 3. Except for week 3, at which the notch incidence was significantly lower in multiparous women (p < 0.001), no additional significant, parity-related differences for notching were detected over time.

The reliability coefficient for the UtA-PI was 0.170. The ICC for absolute agreement among the single observer measurements was 0.985, with a 95% CI ranging from 0.982 to 0.988. Similarly, the reliability coefficient for the UtA-RI measurements was 0.042. The ICC for the absolute agreement among the single observer measurements was 0.982, with a 95% CI ranging from 0.978 to 0.986.

The magnitude of the obtained ICC values for the pulsatility and resistance indices allowed for one of the two measurements obtained from each woman to be ignored in the statistical analysis. Because the sonographer only performed one measurement in his/her day-to-day practice, the first measurements were considered.

### UtA-PI

**Figure 3:**
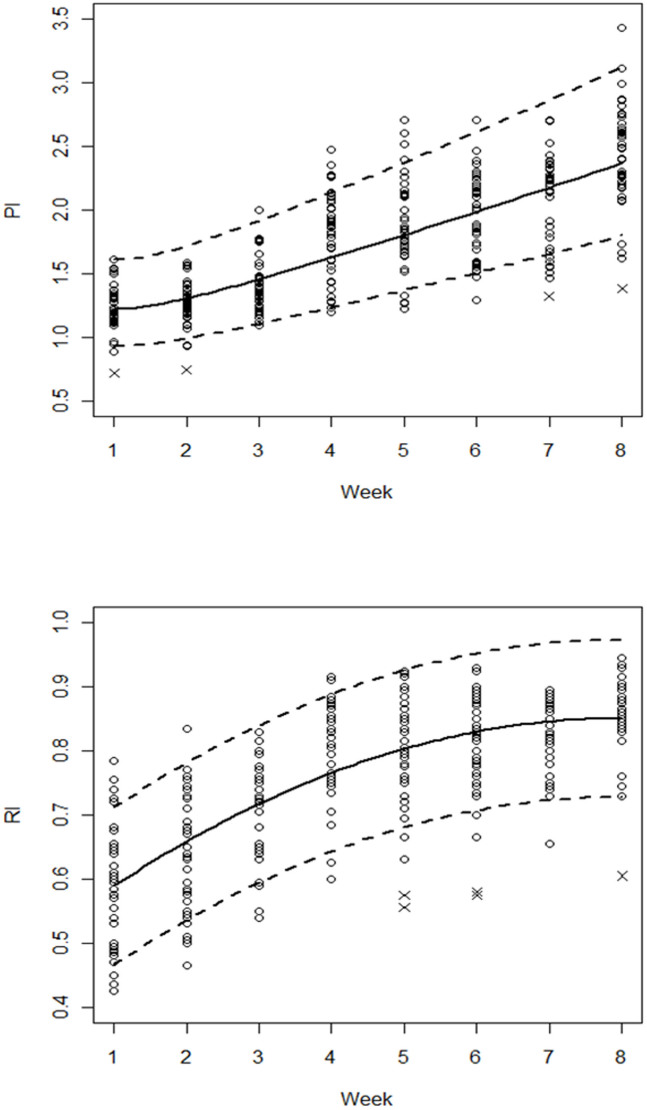
Sample values and the estimated 5^th^, 50^th^ and 95^th^ percentile regression curves for the uterine artery pulsatility (PI) and resistance (RI) indices in all women during the first 8 postpartum weeks. Points marked with (x) were removed from the model fitting to improve normality.

The crude effect of the postpartum week progression on the UtA-PI was estimated by a nonlinear model, which was reduced to a quadratic polynomial on the log-transformed variables. The regression model equation and the estimated coefficient values are presented in Table 4A. The likelihood ratio test favoured the model with no correction for error variances. Due to small departures from normality on the left tail, the 4 observations with standardised residuals less than −2.5 were removed. The UtA-PI values of these women corresponded to the lowest values observed at weeks 1, 2, 7 and 8. The predicted regression curve was basically a concave-up increasing function of the postpartum time, with an increasing rate of softening from the 2^nd^ postpartum week onwards (Figure 3, Table 5).

**Figure 4:**
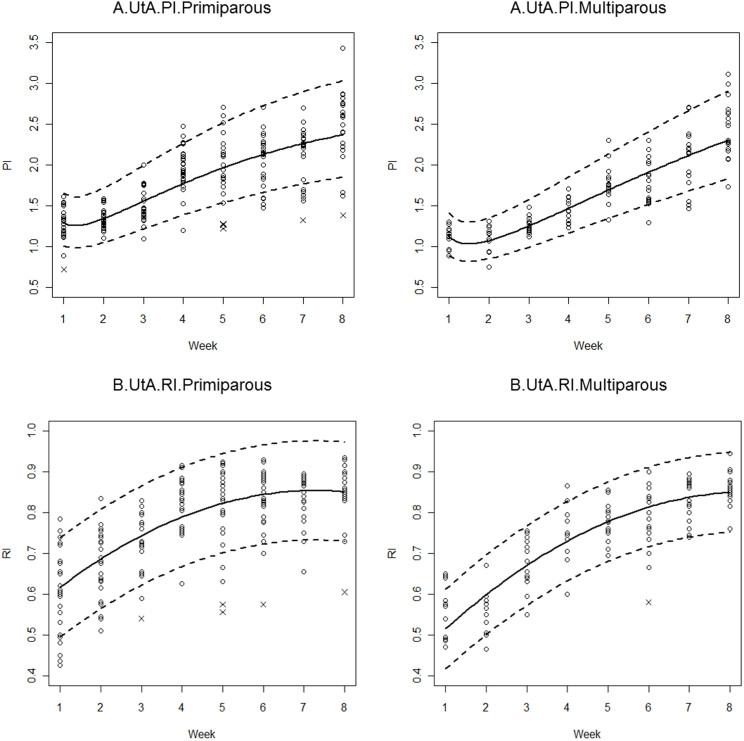
Sample values and the estimated 5^th^, 50^th^ and 95^th^ percentile regression curves for the uterine artery (UtA) pulsatility (A) and resistance (B) indices during the first 8 postpartum weeks, stratified by the parity status (left: primiparous; right: multiparous). Points marked with (x) were removed from the model fitting to improve normality. PI, pulsatility index; RI, resistance index.

Adjustment for potential time effect confounders only identified parity as a statistically significant variable; maternal age, BMI, smoking, mode of delivery, infant birth weight, breastfeeding, and haemoglobin on postpartum day 2 did not have significant effects on the UtA resistance indices during the first eight postpartum weeks (Table 4). The resulting model was a cubic polynomial on the log-transformed variables, with significant second-order interaction terms between parity and postpartum time. Model estimates are presented in Table 4B, and the 5^th^, 50^th^ and 95^th^ percentiles are plotted in Figure 4A. The error variances were allowed to differ depending on the postpartum week. The set of seven observations with standardised residuals less than −2.5 compromised the error normality; therefore, those observations were removed (Figure 4A). As depicted in Figure 4A, multiparous women started the postpartum period by exhibiting lower UTA-PIs than those predicted for the primiparous women. As time progressed, the predicted values for each group approached one another.

### UtA-RI

The crude effect of the postpartum week progression on the UtA-RI was estimated using a quadratic polynomial. The regression model equation and the estimated coefficient values are presented in Table 4A. The likelihood ratio test favoured the model with different variances according to the postpartum week. Due to small departures from normality on the left tail, the 5 observations with standardised residuals less than −2.5 were removed. The UtA-RI values of these women corresponded to the lowest values observed in weeks 5, 6 and 8. The predicted regression curve was a concave-down increasing function, which would theoretically achieve its maximum at 8.6 weeks (Figure 3).

As for the PI, the adjustment for potential time effect confounders only identified parity as a statistically significant variable. The resulting model is presented in Table 4B and is plotted in Figure 4B: it is a quadratic polynomial plotted against postpartum weeks, with different intercept and slope coefficients according to the parity status. Error variances were again allowed to differ with the postpartum week. Six observations with standardised residuals less than −2.5 compromised the error normality and were therefore removed from the model. During the first weeks, the UtA-RI values for multiparous women were significantly lower than those observed for primiparous women (Figure 4, Table 5). However, as time progressed, that effect ceased to be significant, and the predicted values for both groups were no longer significantly different from one another.

## Discussion

During the first few weeks after delivery, an important change occurs in the pelvic circulation because the blood requirements of pregnancy are no longer necessary. Exactly how quickly these regional haemodynamic changes return to the non-pregnant state is not well understood. The process involves spiral artery luminal obliteration due to phenomena such as thrombosis, endarteritis and intima thickening^14^, which result in increased local vascular resistance^15^. Although this finding may reflect systemic circulatory changes^16^,^17^, an immediate cause for the events is the mechanical pressure imposed by the contracted postpartum myometrium, as well as, possibly, local immunological phenomena^18^. As the structural involution of the uterine artery and distal branches occurs, functional changes can be assessed using a computerised analysis of the blood velocity spectrum obtained with Doppler ultrasound. In our cross-sectional investigation, assessment of the UtA-PI and UtA-RI during the first eight weeks postpartum uncovered a progressive, significant increase in UtA impedance.

### Statistics

Our study provides reference ranges of the mean UtA-PI and UtA-RI for each week between 1 and 8 weeks postpartum in an appropriately large sample of healthy, normotensive women. Furthermore, stringent and validated methodological guidelines were employed to construct these reference ranges^19^,^20^. Although a cross-sectional design was used, we included the same number of observations at each week postpartum for reasons related to clinical practice procedures.

The reliability study of the UtA-PI and UtA-RI measurements was based on the ICC. Because the number of obtained coefficients was greater than 0.7, the authors inferred that the measurements were highly repeatable, with a very low measurement error^19^,^21^.

From the statistical point of view, a variety of strategies for constructing reference intervals and percentile charts have been published^22^: linear regression, if necessary, along with modelling of the residual standard deviation; the LMS curves method, introduced by Cole^23^; the non-parametric HRY method of Healy, Rabash and Young^24^; and, finally, non-parametric quantile regression. In this study, the authors used the linear model, regressing UtA-PI and UtA-RI on polynomial functions of the postpartum time and considering adjustments for potential confounders such as parity, maternal age, BMI, smoking, mode of delivery (vaginal vs Caesarean section), infant birth weight, breast-feeding, and haemoglobin on day 2 postpartum. Because the evaluation of the goodness of fit of the model was very satisfactory, the percentile curves for the UtA-PI and UtA-RI were confidently identified from those linear models.

### UtA-PI and UtA-RI reference ranges

Transvaginal assessment of UtA impedance by employing Doppler ultrasound offers several advantages over the transabdominal route^25^. The vessel is easily identified and is located within close proximity, thus yielding clearer waveforms. Additionally, the insonation angle is near 0°, which results in high reproducibility^25^,^26^.

Previous reports addressing postpartum uterine artery impedance reported results with considerable variation in the time-points assessed, the indices employed and the population features, which may underlie the diversity of observations. Although there is a general consensus that within 12–14 weeks postpartum, the uterine artery impedance rises towards the non-pregnant values^13^,^27^,^28^,^29^,^30^,^31^,^32^, the findings are conflicting at earlier days; a marked increase^27^,^28^,^29^,^30^,^31^,^32^, no change or even a minor transient reduction in impedance data have all been reported^13^,^32^.

The current cross-sectional study also provides evidence of an increasing trend towards pre-pregnancy impedance values; in addition, it provides more time points, studied on a regular weekly basis, and considerably extended the number of patients. In agreement with a previous longitudinal study^13^,^33^ this study showed that by 8 weeks postpartum, the regional pelvic circulation had not yet recovered to the non-pregnant state^27^, emphasising that the time needed for UtA re-adaptation to the non-pregnant state is more extensive than previously assumed^13^,^27^. It is possible that longer-term factors acting at the endothelium of the uterine artery or systemically^6^,^31^ take part in the cardiovascular adaptation that results in the normal waveform^30^.

Accompanying this trend, the reappearance of the proto-diastolic notch was noticed as early as the second day postpartum^27^. Our study confirms that in the early days after delivery, only a small number of patients will display notching (defined as at least one notch) but, with a gradual increase each week, the presence of this feature approaches 100% of cases, similarly to the findings of a previous report from others^13^.

### Effect of parity on uterine flow impedance

In our research, maternal age, BMI, smoking, mode of delivery (vaginal vs Caesarean section), infant birth weight, breast-feeding, and haemoglobin on day 2 postpartum did not cause any significant effects on UtA impedance during the first eight weeks postpartum. However, during the same period, UtA impedance was significantly affected by maternal parity, a condition not examined in previous reports and a likely reason for the diversity of results.

In the current study, multiparous women exhibited lower UTA PIs and RIs during the early weeks, which converged to values similar to primiparous RIs and PIs by the eighth week. Presumably, the UtA capacitance properties observed in multiparous women are the result of vascular structural features that remained from the first pregnancy. In fact, although the well-known spiral artery remodelling that occurs during pregnancy^34^ reverts upon delivery, not all vessels recover to their pre-pregnancy conditions. Indeed, spiral artery internal elastic lamina duplication or fragmentation was described at the endometrial/myometrial junction of parous women in contrast to nulliparous women^35^. We are convinced that such permanent structural changes endow the spiral arteries with reduced impedance and underlie the parity-related lower UtA-PI and UtA-RI values that we report in the immediate postpartum period.

### External validity, study limitations and future research

As with any study, the choice of an appropriate sample is of great importance. While some published studies used routinely collected data, resulting in the inclusion of multiple observations of some participants, Altman and Chitty^36^ advocate specifically collecting data for the purpose of developing reference ranges (sometimes misleadingly called ‘normal ranges’), with each participant being included only once. Within this objective, it is important to have as unselected a sample as possible because reference data should relate to ‘normal’ characteristics^37^. In reality, ‘reference intervals’ for known sonographic features, either maternal or foetal, are not synonymous with ‘normal ranges’ for these characteristics^36^,^37^. This is because in clinical practice, it is extremely difficult (nearly impossible) to obtain a truly unselected population. Our goal was achieved with a methodology that sought an appropriate selected sample, but we did not have a truly unselected population. Therefore, any maternal condition that could potentially affect the uterine artery Doppler was deemed reasonable to be an exclusion criterion. However, our study design may attract criticism for producing supernormal ranges that are less applicable to the general population. For this reason, despite the current inclusion criteria, we did not exclude participants due to complicating factors that developed after recruitment. Nevertheless, no complications occurred in the studied group (patients ‘Included in the study’) during the first eight postpartum weeks.

As with the introduction of any new technology into clinical practice, it is essential that those conducting Doppler assessment are adequately trained and that their results are subjected to rigorous audit. Although our data were collected by a single, very experienced operator, which could compromise the external validity of his results, Doppler blood flow measurements of the UtA impedance were found to be highly repeatable only when carried out by well-trained operators^25^. Because the usefulness of a screening test depends not only on its predictive ability but also on its reproducibility, future studies are needed to demonstrate the usefulness of these reference ranges, as well as their applicability.

In short, the median 5^th^ and the 95^th^ percentile regression curves of the pulsatility and resistance indices of the uterine artery increase from week 1 to week 8 postpartum. Parity does not change the trend, but primiparous women have higher UtA resistance indices in the earlier postpartum weeks. The UtA impedance reference ranges presented here are likely to be clinically useful in the assessment of postpartum disorders, including retained placental tissue in the uterine cavity, which delays the normal involution of uterine vessels^18^,^35^, infection, postpartum bleeding and enhanced myometrial vascularity^15^. Additional studies are necessary to confirm these findings and to further support the use of postpartum UtA Doppler patterns as a prognostic tool.

## Methods

This study was approved by the local ethics committee of the Hospital Centre of Porto, Department of Women's Reproductive Medicine. All of the subjects provided their informed consent (IRB protocol number: 150-13[096-DEFI/122-CES]). The methods were carried out in accordance with the approved guidelines.

From January 2010 to December 2013, a prospective, cross-sectional, observational study was conducted, including 320 healthy women with singleton pregnancies who gave birth between the 37^th^ and 41^st^ gestational week, either vaginally or by Caesarean section. Gestational age was calculated by ultrasonography performed between 11 and 14 weeks, and the participants were consecutively recruited after meeting the eligibility criteria.

Each woman was assessed only once between days 1 and 56 postpartum and was categorised to the respective week to assess a total of 40 women per week. During the postpartum appointments, a senior specialist reviewed the patients' medical histories and verified the absence of hormonal contraception or intrauterine devices, present or past gynaecological pathology (e.g., fibroids, adnexal pathology or prior gynaecologic surgery), hypertension, diabetes and other endocrine disorders, immune diseases, renal and structural heart diseases, haematological conditions (with particular emphasis on postpartum haemorrhage) and acute or chronic infections. Current acceptable medications included vitamin and iron supplements; previous administration of prostaglandin E analogues, low-dose oxytocin regimens, and vaginal misoprostol to induce labour in accordance with ACOG Practice Bulletin N.107 were also acceptable^38^.

The medical records included each patient's age, body mass index (BMI), parity, pregnancy outcome, mode of delivery, and haemoglobin level on day 2 postpartum, the child's birth weight and Apgar score, and whether the mother was breast- or formula-feeding.

Prior to uterine artery Doppler evaluation, we conducted a rigorous transvaginal ultrasound to exclude uterine structural defects, retained placental tissue or adnexal pathology. In the first week after delivery, a transabdominal assessment complemented the evaluation to improve the accuracy. In this setting, the presence of fluid and debris in the uterine cavity was considered to be irrelevant^39^. Doppler flow studies were consecutively scheduled until the requirement of 40 measurements per postpartum week was met.

### Doppler flow study

UtA Doppler examinations were performed using a Voluson 730 Pro (GE Healthcare Technologies, Milwaukee, WI, USA) with a 5-MHz vaginal probe. Employing a transvaginal transducer (GE Healthcare Probe Type RIC5-9 W, Horizontal Standard EN60601-1), a single experienced operator (LG-M; 6 years of experience in obstetric and gynaecologic ultrasound) made all of the measurements to minimise inter-observer variability. The measurements were made with the women in the lithotomy position and with empty urinary bladders. Smokers and nursing mothers were required to abstain from smoking and breastfeeding for at least 2 h and 30 minutes, respectively, before the procedure.

A sagittal section of the uterus was obtained, and the cervical canal and internal cervical ostium were identified. Subsequently, the transducer was gently tilted from side to side, and then colour flow mapping was used to identify each uterine artery along the side of the cervix and uterus at the level of the internal ostium. Pulsed wave Doppler was used with the sampling gate set at 1–2 mm to cover the entire vessel, and angle correction was used where appropriate to ensure that the angle of insonation was less than 30°. The PI and RI were measured when three similar consecutive waveforms were obtained; then, the mean PI and RI of the left and right arteries were calculated (Figure 2). In general, no more than 1 minute was needed to assess both arteries. We noted the presence or absence of an early bilateral protodiastolic notch, defined as a persistent decrease in blood flow velocity in early diastole below the diastolic peak velocity.

Intra-observer reliability was obtained from two consecutive readings at the beginning and the end of the scan of the 640 recordings (right UtA 320 + left UtA 320) of resistance and pulsatility indices in the uterine arteries. In the present context, there was only a single observer, who was completely unaware of any of the other results.

### Efforts to address potential sources of bias

Any postpartum women attended by our clinical investigator during the study protocol were considered to be potentially eligible. The scheduling of postpartum consultations was randomly made by the hospital secretariat according to the availability of the clinical investigator (AC). Patients were consecutively recruited, and ultrasonographic examinations were scheduled so that a total of 40 women were examined per postpartum week. The selection of the participants was achieved by a single investigator who was unaware of the Doppler measurement results. Finally, the researcher responsible for the Doppler measurements had no access to clinical data that was obtained by the investigator responsible for determining patient eligibility.

### Statistical analysis

Sample size calculation basically followed Altman & Ohuma (2013)^40^ and Papageorghiou *et al.* (2014)^41^. It was carried out in relation to the precision and accuracy of a single percentile and regression-based limits, as first proposed by Royston (1991)^42^ and extended by Bellera & Hanley (2007)^43^. In 1991, Royston^42^ provided the following formula for the standard error of a *p*th percentile from the normal distribution:
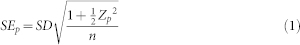
where *SE* is the standard error, *SD* is the standard deviation of the measurements, *Z_p_* is the value of the standard normal distribution corresponding to the *p*th percentile, and *n* is the sample size.

Our aim was to obtain the 5^th^ and the 95^th^ reference percentiles, with *Z_p_* = 1.645. A cross-sectional study of 320 individuals, with 40 individuals sampled per week, would then provide
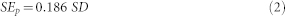
Bearing in mind that the distance between the extreme percentiles is approximately 3.3 SD, the equation above shows that the percentile estimates will be given with great precision.

Bellera & Hanley^43^ showed that the above formula is only valid at the mean value of the time covariate. Assuming that the postpartum weeks are uniformly distributed, our sample size would provide a relative margin of error (ratio of the width of the confidence interval for the reference limit to the width of the reference range) of approximately 13%, which also seems to be very reasonable. Nevertheless, as stated by Bellera & Hanley^43^, whenever heteroscedasticity of PI values across postpartum time exists, the above formulae should be used as a rough guide for sample size planning.

Intra-class correlation coefficients (ICC) and 95% confidence intervals (CIs) were calculated with a two-way mixed-effects model. The reliability coefficient, which is the difference value that will be exceeded by only 5% of pairs of measurements on the same subject, was calculated as 1.96 times the standard deviation (SD) of the difference between pairs of repeated measurements^19^. The chi-square test or Fisher's test (as appropriate) was used to compare proportions.

Maternal age, BMI, parity status (primiparous vs multiparous), smoking, mode of delivery (vaginal vs Caesarean section), infant birth weight, breastfeeding, and haemoglobin on day 2 postpartum were considered potential time-effect confounders. However, adequate adjustment for these variables identified parity as the only statistically significant confounder.

Population reference intervals for the crude and parity status-adjusted behaviour of PI and RI during the first eight postpartum weeks were derived from regression models^20^. The fitting process utilised generalised least squares, allowing for errors with unequal variances. Only linear models were considered, occasionally with log-transformed variables, and no polynomials of a degree greater than 3 were used. In fact, these curves can exhibit unrealistic features such as waviness or sharp deviations at extreme values of the domain^20^. After removing the outliers (no more than 7 within each model), the error distribution assessment did not compromise normality. The 5^th^ (resp. 95^th^) percentile curve was then given by the regression predicted curve minus (resp. plus) 1.96 times the residual standard deviation, whereas the 50^th^ percentile curve corresponded to the (mean) regression curve. Whenever possible, model comparisons were based on the likelihood ratio test; otherwise, they were based on the Bayesian information criterion (BIC).

The crude (resp. parity adjusted) effect of the postpartum week progression on the UtA-PI was estimated by a nonlinear model that reduced to a quadratic (resp. cubic) polynomial on the log-transformed variables. The crude (resp. parity adjusted) effect of the postpartum week progression on the UtA-RI was estimated by a quadratic polynomial on the original variables. All of the models, except that of the effect of the crude time on UtA-PI, presented different error variances according to the week being considered.

All of the statistical analyses were performed using the R language and software environment for statistical computation, version 2.12.1^44^. The significance level was fixed at 0.05.

Our research has adhered to the Strengthening the Reporting of Observational studies in Epidemiology (STROBE) guidelines for observational studies, and all recommendations were included in the study.

## Author Contributions

L.G.-M. designed the study, performed all Doppler measurements, analysed the data, and wrote the manuscript. A.R.G. performed all statistical analyses. J.S. contributed to the critical revision of the manuscript. A.C. coordinated the review of clinical cases. F.M. designed the study. H.A. designed the study, analysed the data, and wrote the manuscript. All authors contributed to the data interpretation and the final version of the manuscript, which all approved. All authors read and approved the final manuscript.

## Figures and Tables

**Table 1:** Main characteristics and obstetric data of the 320 women included in the study

		n (%)
Age intervals, in years	18–24	54 (16.9)
	25–34	166 (51.9)
	35–43	100 (31.2)
Age, *years* (mean ± SD*)	30.8 ± 6.50	-
Ethnicity	White	309 (96.6)
	Black	4 (1.2)
	Other	7 (2.2)
Parity	>1	119 (37.2)
Body mass index† (*kg/m^2^*)	16–24	118 (36.9)
	25–29	127 (39.7)
	30–44	75 (23.4)
Smoking†		53 (16.6)
GA‡ at delivery, *weeks* (median ± IQR**)	40.1 (39.1–40.6)	-
Caesarean section		129 (40.3)
Apgar score at 5′	<7	0 (0)
Birth weight at delivery, *g* (mean ± SD*)	3160.7 (±344.93)	-
Haemoglobin on day 2, *g/dL* (mean ± SD*)	11.1 (±1.05)	-
Breastfeeding†		223 (69.7)

*SD, standard deviation;

**IQR, interquartile range;

^†^Obtained by the moment of the Doppler flow acquisition;

^‡^GA, gestational age.

**Table 2:** Indication for Caesarean sections (*n* = 129) in the study sample

		Caesarean Deliveries (%)*
Primary	Dystocia	26 (20.2)
	Non-reassuring foetal heart rate	17 (13.2)
	Abnormal presentation	15 (11.6)
	Unsuccessful trial of forceps or vacuum	12 (9.3)
Repeat	No VBAC attempt	38 (29.5)
	Failed VBAC	14 (10.8)
	Unsuccessful trial of forceps or vacuum	7 (5.4)

*Data are shown as absolute (relative, %) frequencies; VBAC, vaginal birth after caesarean.

**Table 3:** Absolute (relative, %) parity frequencies in each group of 40 women examined per week and absolute (relative, %) frequencies for postpartum uterine artery positive notching (at least one notch)

	Parity		Positive Notching				
Week	Primiparous	Multiparous	All n (%)	p-value*	Primiparous n (%)	Multiparousn (%)	p-value†
1	28 (70.0)	12 (30.0)	9 (22.5)	<0.001	8 (28.6)	1 (8.3)	0.233
2	28 (70.0)	12 (30.0)	8 (20.0)		8 (28.6)	0 (0)	0.079
3	26 (65.0)	14 (35.0)	17 (42.5)		16 (61.5)	1 (7.1)	<0.001
4	28 (70.0)	12 (30.0)	31 (77.5)		25 (89.3)	6 (50.0)	0.012
5	24 (60.0)	16 (40.0)	34 (85.0)		21 (87.5)	13 (81.2)	0.668
6	24 (60.0)	16 (40.0)	32 (80.0)		20 (83.3)	12 (75.0)	0.691
7	22 (55.0)	18 (45.0)	35 (87.5)		21 (95.5)	14 (77.8)	0.155
8	21 (52.5)	19 (47.5)	38 (95.0)		20 (95.2)	18 (94.7)	1.000

*p-value from a *χ*^2^-test assessing the value of positive notching frequencies along the postpartum weeks;

^†^p-value from *χ*^2^-tests assessing the homogeneity of proportions between primiparous and multiparous women (using Bonferroni's correction for multiple comparisons, significance should be taken at the level of 0.006 [0.05/8]).

**Table 4:** Estimates of the regression coefficients and corresponding p-values for the PI and RI models during the postpartum period (A), as well as stratified by the parity status (B). The additive effects of potential time confounders are also shown; only the parity status had a statistically significant time-adjusted effect (A)

		Variables	Regression Coefficients	p-values
A*	log (PI)	*Intercept*	0.203	0.000
		*log*(*Week*)	−0.023	0.653
		*log*^2^(*Week*)	0.164	0.000
		*Multiparous (vs primiparous)*	−0.127	0.000
		*BMI 25–29 (vs BMI 16–24)*	−0.032	0.137
		*BMI 30–44 (vs BMI 16–24)*	−0.025	0.337
		*Age 25–34 (vs age 18–24)*	−0.022	0.419
		*Age 35–43 (vs age 18–24)*	0.001	0.974
		*Vaginal (vs Caesarean)*	0.036	0.061
		*Smoker (vs non-smoker)*	0.017	0.506
		*Birth weight (g)*	0.000	0.991
		*Haemoglobin on day 2 (g/dL)*	−0.007	0.470
	RI	*Intercept*	0.508	0.000
		*Week*	0.086	0.000
		*Week*^2^	−0.005	0.000
		*Multiparous (vs primiparous)*	−0.037	0.000
		*BMI 25–29 (vs BMI 16–24)*	0.002	0.819
		*BMI 30–44 (vs BMI 16–24)*	0.002	0.873
		*Age 25–34 (vs age 18–24)*	−0.002	0.860
		*Age 35–43 (vs age 18–24)*	0.001	0.940
		*Vaginal (vs Caesarean)*	0.001	0.943
		*Smoker (vs non-smoker)*	−0.008	0.416
		*Birth weight (g)*	0.694	0.694
		*Haemoglobin on day 2 (g/dL)*	0.106	0.106
B†	log (PI)	*Intercept*	0.255	0.000
		*Multiparous (vs primiparous)*	−0.141	0.001
		*log*(*Week*)	−0.229	0.019
		*log*(*Week*): *Multiparous*	−0.209	0.018
		*log*^2^(*Week*)	0.489	0.000
		*log*^2^(*Week*): *Multiparous*	0.127	0.002
		*log*^3^(*Week*)	−0.115	0.003
	RI	*Intercept*	0.535	0.000
		*Multiparous (vs primiparous)*	−0.117	0.000
		*Week*	0.088	0.000
		*Week: Multiparous*	0.014	0.000
		*Week*^2^	−0.006	0.000

*A: The estimated equation for PI was 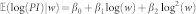 or, equivalently, 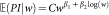 with 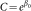, for any given week *w*. The estimated equation for RI was 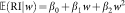.

^†^B: The estimated equation for PI was 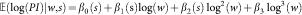 or, equivalently, 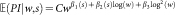 with 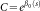, for any given week *w* and parity status *s*. The estimated equation for RI was 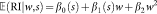.

The letter 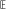 in the equations denotes the expected value. The primiparous category was taken as the reference class. PI, pulsatility index; RI, resistance index; UtA, uterine artery.

**Table 5:** Predicted percentile values for the uterine artery pulsatility (PI) and resistance (RI) indices according to postpartum week; A - not accounting for parity status; B - stratified by parity status

A						
	PI			RI		
Weeks	5^th^	50^th^	95^th^	5^th^	50^th^	95^th^
1	0.930	1.225	1.613	0.466	0.589	0.711
2	0.990	1.304	1.717	0.536	0.659	0.781
3	1.104	1.454	1.915	0.595	0.718	0.840
4	1.233	1.624	2.139	0.643	0.766	0.889
5	1.368	1.802	2.373	0.681	0.803	0.926
6	1.508	1.986	2.615	0.707	0.830	0.953
7	1.651	2.174	2.864	0.723	0.846	0.968
8	1.797	2.367	3.117	0.728	0.851	0.973

B						
	Primiparous			Multiparous		
Weeks	5^th^	50^th^	95^th^	5^th^	50^th^	95^th^
PI						
1	1.008	1.291	1.653	0.889	1.121	1.413
2	1.047	1.341	1.717	0.849	1.071	1.350
3	1.215	1.556	1.992	0.992	1.251	1.578
4	1.384	1.772	2.269	1.166	1.470	1.853
5	1.534	1.965	2.516	1.343	1.693	2.134
6	1.662	2.128	2.725	1.515	1.910	2.408
7	1.767	2.262	2.897	1.677	2.115	2.666
8	1.850	2.369	3.033	1.829	2.306	2.907
RI						
1	0.495	0.617	0.738	0.417	0.514	0.612
2	0.565	0.686	0.808	0.501	0.598	0.696
3	0.622	0.744	0.865	0.573	0.670	0.768
4	0.668	0.789	0.911	0.632	0.730	0.828
5	0.701	0.823	0.944	0.680	0.778	0.875
6	0.722	0.844	0.966	0.716	0.814	0.911
7	0.732	0.854	0.975	0.740	0.837	0.935
8	0.729	0.851	0.972	0.751	0.849	0.947
